# Current progress in the derivation and therapeutic application of neural stem cells

**DOI:** 10.1038/cddis.2017.504

**Published:** 2017-10-12

**Authors:** Yuewen Tang, Pei Yu, Lin Cheng

**Affiliations:** 1 National Research Center for Translational Medicine, State Key Laboratory of Medical Genomics, Shanghai Institute of Haematology, Rui Jin Hospital Affiliated to Shanghai Jiao Tong University School of Medicine, Shanghai, China; 2 Department of Orthopaedics, Rui Jin Hospital Affiliated to Shanghai Jiao Tong University School of Medicine, Shanghai, China

## Abstract

Neural stem cells (NSCs) have a unique role in neural regeneration. Cell therapy based on NSC transplantation is a promising tool for the treatment of nervous system diseases. However, there are still many issues and controversies associated with the derivation and therapeutic application of these cells. In this review, we summarize the different sources of NSCs and their derivation methods, including direct isolation from primary tissues, differentiation from pluripotent stem cells and transdifferentiation from somatic cells. We also review the current progress in NSC implantation for the treatment of various neural defects and injuries in animal models and clinical trials. Finally, we discuss potential optimization strategies for NSC derivation and propose urgent challenges to the clinical translation of NSC-based therapies in the near future.

## Facts

- NSCs are a promising treatment modality for diseases associated with the nervous system as they secrete soluble factors and differentiate into neurons, astrocytes and oligodendrocytes.
- NSCs can be derived from three different sources using recent technical advances: direct isolation from primary tissues, differentiation from pluripotent stem cells and transdifferentiation from somatic cells.
- Cell therapies based on NSC transplantation for the treatment of various neural defects and injuries in animal models and clinical trials have been widely investigated.

## Open Questions

- Which NSC derivation strategy is most efficient and safe for clinical translation?
- How can NSC transplantation methods be translated from preclinical studies into clinical trials?
- What are the optimization strategies and urgent challenges for the clinical translation of NSC-based therapies in the near future?

**Figure 1:**
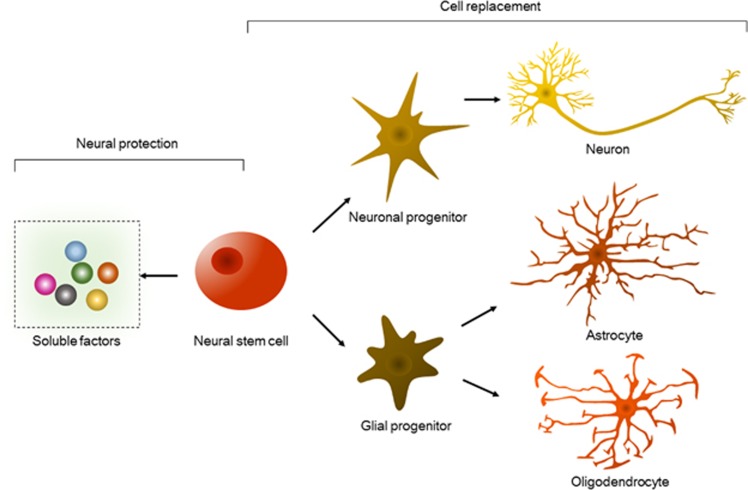
NSC properties for therapeutics. NSCs secrete soluble factors, including neurotrophic factors, growth factors and cytokines, thus protecting existing neural cells against damage *in situ*. Furthermore, they differentiate into neurons, astrocytes and oligodendrocytes via committed progenitor stages to replace lost neural cells. Either neural protection or cell replacement may aid in neurological functional recovery after acute or chronic injury via neural regeneration

Nervous system diseases are refractory diseases that can cause loss of sensation, loss of motor function and memory failure, as well as directly threaten the life of a patient. Currently, the pathogenic factors involved in these diseases and their pathogenesis are unclear. Traditional drug treatments are used to delay disease progression and cannot restore function or regenerate tissues. Recent studies have indicated that the transplantation of neural stem cells (NSCs) is a promising treatment modality for diseases associated with the nervous system, for the regeneration of neural cells and for the restoration of the microenvironment at the injury site (Figure 1).

**Figure 2:**
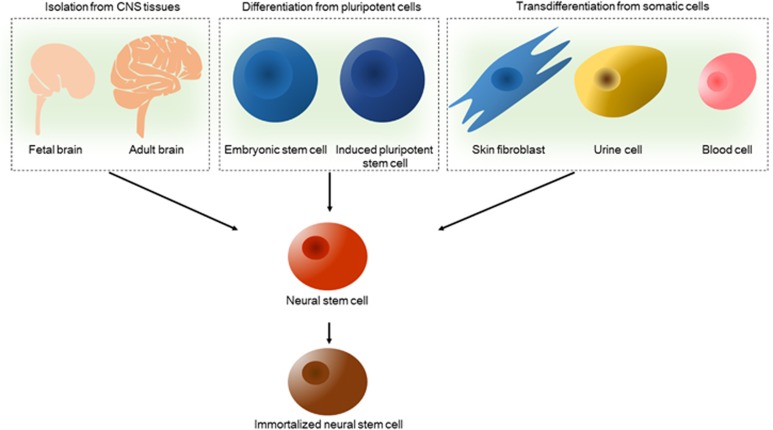
Sources of NSCs. Using recent technical advances, NSCs can be derived via three diverse methods: direct extraction from primary CNS tissues, including fetal brain, adult brain and spinal cord tissue; differentiation from pluripotent stem cells, such as embryonic stem cells and induced pluripotent stem cells; and transdifferentiation from somatic cells, such as skin fibroblasts, urine cells and blood cells, which are easily harvested in the clinic. NSCs generated from the above sources can be further immortalized via genetic modification

The cell source is the first issue that must be addressed to enable the application of NSCs in clinical treatments because the cell dose required for adequate transplantation is very high. NSCs can be derived from three different sources using recent technical advances, including direct extraction from primary tissues, differentiation from pluripotent stem cells and transdifferentiation from somatic cells (Figure 2 and Table 1).

## Strategies for the Isolation and Generation of NSCs

### Isolating and culturing NSCs from primary tissues

The establishment of cell isolation and cell culture techniques has led to the development of favorable experimental methods and the identification of a rich cell source for NSC research. In 1992, Reynolds and Weiss isolated NSCs from the striatum of the adult mouse brain and reported the first use of epidermal growth factor (EGF) to induce NSC proliferation *in vitro*.^1^ Two years later, they found that the subependymal region in the mouse brain is the source of NSCs *in vivo*.^2^ Based on previous results, Weiss further reported that EGF and basic fibroblast growth factor (bFGF) cooperatively induce the proliferation, self-renewal and expansion of NSCs isolated from adult mouse thoracic spinal cord.^3^ NSCs can grow in single-cell suspensions obtained by enzymatic digestion and form spherical clusters called neurospheres, which are non-adherent and can be re-plated in selective culture medium to obtain neural cells.^4, 5^ Neurospheres can also be sub-cultured to expand the pool of NSCs for experimental or therapeutic purposes. Periventricular regions and olfactory bulb in adult mammalian brains are rich sources of NSCs.^6, 7, 8^ Beyond these methods of isolating NSCs through diverse culturing strategies, NSCs can also be directly isolated by cell sorting based on the expression of NSC surface markers.^9^ Belenguer and Guo have developed an optimized protocol for the isolation, culture and expansion of NSCs from mammalian animals.^10, 11^ Although, a canonical protocol for obtaining human tissue-derived NSCs has not yet been established, the technical methods are generally similar to the ones applied in animals, and the tissues must be obtained in accordance with ethical guidelines.

### Differentiation of pluripotent stem cells into NSCs

Pluripotent stem cells, including embryonic stem cells (ESCs) and induced pluripotent stem cells (iPSCs), can generate desired cells through differentiation, which are attractive alternatives to primary cell isolation. Generally, protocols for neural differentiation from pluripotent stem cells can be categorized into two major routes: embryoid body (EB) formation and monolayer culture.

In the protocol for EB formation, ESC and iPSC colonies are detached and grow in suspension to form EBs. Subsequently, EBs are plated on adhesive substrates with defined serum-free medium that promote the generation of neural tube-like rosettes and the selection of neural progenitor cells (NPCs).^12^ Zhang *et al.*^13^ first described the differentiation, enrichment and transplantation of neural precursor cells from human ESC-derived EBs *in vitro*. Later, Kozhich *et al.*^14^ developed a novel protocol suitable for standardized generation and differentiation of neural precursor cells from human pluripotent stem cells, making iPSC-derived NSCs an appealing source for cell-based therapies.^15^ Pluripotent stem cells can also directly differentiate into NSCs in monolayer culture via the neural rosettes stage, which is a striking feature here.^16, 17, 18^ A serum-free and nutrient-poor medium is utilized to initiate differentiation, and depending on the cell line, additional growth factors or inhibitors may be required to promote neural differentiation.^19^ Banda and Grabel^20^ have been focusing on using monolayer culture to directly differentiate human ESCs into neural progenitors, which includes four typical stages. Wen and Jin^21^ have developed a straightforward and useful strategy for the generation of NSCs from both human ESC and iPSC lines. Comparisons of NSC marker expression and morphology have indicated that there are no significant differences between the NSCs derived via EB formation and monolayer culture methods.

### Transdifferentiation of somatic cells into NSCs

The term transdifferentiation, also known as lineage reprogramming, was originally coined by Selman and Kafatos in 1974.^22^ During this process, one type of mature somatic cell transforms into another type of mature somatic cell without undergoing an intermediate pluripotent state.^22, 23^ This process is induced mainly by the exogenous expression of lineage-specific transcription factors (TFs) and by chemical compounds.

### TF-induced transdifferentiation

Ding and others first demonstrated that transient expression of pluripotency factors combined with the appropriate neural signaling inputs can successfully induce mouse fibroblasts to form expandable NSCs,^24, 25^ which were called induced NSCs (iNSCs). This finding provides a new strategy for the generation of NSCs through direct cell transdifferentiation following virus-mediated exogenous gene expression. Aside from the pluripotency factors, NSC-specific TFs can also induce the generation of neural stem-like cells with self-renewal and tripotent differentiation potential.^26, 27^ Furthermore, it is found that a single TF, *Sox2* or *ZFP521*, can be used to generate iNSCs from mouse and human fibroblasts.^28, 29^ Two Chinese teams, led by Zhang and Ding, have separately combined defined TFs with chemical cocktails that enable the generation of expandable iNSCs from both primate and human fibroblasts,^30, 31^ thus suggesting that small-molecule chemicals can increase the efficiency of iNSC generation. In addition to fibroblasts, many other somatic cell types are considered to be ideal starting cells for NSC generation depending on the clinical situation, including Sertoli cells,^32^ adult liver cells and B lymphocytes,^33^ urine epithelial-like cells,^34^ astrocytes,^35^ and cord blood sample.^36^

### Chemical compound-induced transdifferentiation

In recent years, researchers have explored chemical reprogramming as a new method to manipulate cell fate. Compared with the conventional practice of importing exogenous viral genes to induce cell transdifferentiation, the use of small-molecule chemicals to elicit cell transdifferentiation has many obvious advantages in terms of safety and controllability.

We were the first to report using only a chemical cocktail containing valproic acid (VPA), CHIR99021 and Repsox, which inhibits histone deacetylases (HDACs), glycogen synthase kinase (GSK)-3 and transforming growth factor (TGF)-*β*, respectively, to generate chemically induced NPCs (ciNPCs) from mouse embryonic fibroblasts under hypoxic conditions.^37^ Further assays confirmed that mouse tail-tip fibroblasts and human urinary cells can also be induced into ciNPCs via treatment with the same chemical cocktail. This work demonstrates that direct lineage-specific conversion to NPCs can be achieved without introducing exogenous genes and that physiological hypoxia is essential for the initial transition process. In the absence of hypoxic conditions, Zhang *et al.*^38^ developed a cocktail of eight small-molecule components, namely CHIR99021, LDN193189 (an inhibitor of the BMP type I receptor ALK2/3), A83-01 (an inhibitor of the TGF-*β* type I receptor ALK4/5/7), Hh-Ag1.5 (a potent smoothened agonist), RA, SMER28 (an autophagy modulator), RG108 (a DNA methyltransferase inhibitor) and Parnate (a histone demethylase inhibitor), which can efficiently and specifically transdifferentiate mouse fibroblasts into NSC-like cells. These cells resemble primary NSCs in terms of their long-term self-renewal and tripotent differentiation abilities. Takayama *et al.*^39^ also developed a small-molecule cocktail composed of VPA, Forskolin (an adenylyl cyclase activator), Parnate, CHIR99021, Repsox, Dorsomorphin (a selective inhibitor of BMP signaling) and SB431542 (a selective inhibitor of TGF-*β* receptor I, such as ALK4 and ALK7) to induce neural crest-like precursors from mouse embryonic fibroblasts. Zheng *et al.*^40^ showed that a combination of A83-01, Purmorphamine (a smoothened receptor agonist), VPA and Thiazovivin (a selective Rho-associated protein kinase inhibitor) can directly lead to the generation of ciNSCs. Despite these achievements, the mechanisms underlying chemically induced transdifferentiation remain largely unknown. Global gene expression profiles determined through microarray analysis have revealed that the small-molecule-based culture method strongly affects cell identity and specifically induces neural differentiation and development-related genes. Interestingly, small molecules targeting HDACs, GSK-3 and TGF-*β* are included in most of the abovementioned reports and may constitute the core chemicals. It can be speculated that HDAC inhibitors cause chromatin decondensation and induce cells into a plastic state, TGF-*β* inhibitors may regulate cell transition between the mesenchymal and epithelial states and promote cell fate conversion, and GSK-3 inhibitors probably activate Wnt signaling, which helps maintain stem cell properties.^41^

### Growth factor or three-dimensional culture-induced transdifferentiation

Regardless of the method used for NSC derivation, growth factors are utilized, which indicate the significance of these factors. Without the introduction of any exogenous genes and chemicals, Feng *et al.*^42^ successfully established a three-step induction protocol that generates highly purified neural stem-like cells from human adipose-derived mesenchymal stem cells (MSCs) by activating *SOX1* with conditional medium, EGF and bFGF. In addition, Song and Sanchez-Ramos^43^ proposed a detailed protocol with which to generate neural-like progenitors from bone marrow-derived MSCs and umbilical cord blood-derived MSCs. Later, Ge *et al.*^44^ found that cerebrospinal fluid containing growth factors may be a better microenvironment for a more rapid transition of MSCs to a NSC fate. Recently, Gao *et al.*^45^ reported a method for neural precursor cell generation from mouse fibroblasts using physical stress and a few growth factors, including EGF, bFGF, leukemia inhibitory factor and heparin, which synergize to regulate the signaling pathways upstream and downstream of Sox2. During this direct induction process, cells first pass through a transient partially reprogrammed state, and then, cell transdifferentiation is achieved via a safe, non-integrated and efficient method.

Given that stem cells reside in specific niches *in vivo*, a three-dimensional *in vitro* culture system should mimic the complex physical environment and enhance NSC self-renewal and multipotency compared with traditional two-dimensional culture conditions. Su *et al.*^46^ reported that mouse fibroblasts can be converted into three-dimensional spheres on non-adherent substrates and later exhibit the characteristics of neural progenitor-like cells in terms of cell morphology, specific marker expression and self-renewal ability. Immunocytochemical experiments have indicated that the expression of *Sox2* is significantly upregulated in three-dimensional cultured mouse fibroblasts. This study has introduced a new paradigm for safer and more convenient cell transdifferentiation using physical tools. This study suggests that other three-dimensional scaffolds might also be used during iNSC generation. For example, graphene foam, a three-dimensional porous scaffold that is biocompatible and conducive to NSC proliferation, has shown great potential for NSC research, neural tissue engineering and neural prostheses.^47^

## Progress in NSC Transplantation for the Treatment of Diseases

### Neurodegenerative diseases

Neurodegenerative diseases are caused by neural or glial cell defects in the brain or spinal cord, which lead to memory deterioration, cognitive disorders, dementia or body movement disorders and mainly include amyotrophic lateral sclerosis (ALS), Parkinson’s disease (PD), Alzheimer's disease (AD) and Huntington’s disease (HD).

ALS is characterized by degeneration and loss of motor neurons in the cerebral cortex, brain stem and spinal cord, thus resulting in muscle wasting, weakness and, eventually, death within 5 years.^48^ Human NSCs secrete glial cell line-derived neurotrophic factor and brain-derived neurotrophic factor, which induced the regeneration of motor neurons in a transgenic rat model of ALS.^49^ Besides, human iPSCs-derived NSCs effectively improve the function of neuromuscular and motor units and significantly increase the lifespan of ALS mice after intrathecal or intravenous injection.^50^ Beyond the animal models, NSC treatment for ALS has been at the clinical trial stage for years. The clinical results indicate the safety of this therapeutic approach via spinal cord injection.^51, 52, 53^

PD is a disease characterized by the loss of dopamine neurons in the substantia nigra pars compacta and their terminals in the striatum.^54^ In toxin-induced animal models of PD, transplanted human NSCs stimulate the dedifferentiation of rat astrocytes and the secretion of exogenous growth factors, thus inhibiting the activation of microglial cells and slowing PD progression by modulating the lesion microenvironment.^55, 56^ As Lmx1a contributes to NSC differentiation into dopamine neurons, the transplantation of *Lmx1a*-overexpressing iNSCs markedly enhances the efficiency of dopamine neuron production and elicits therapeutic effects in a PD mouse model.^57^ Although NSC transplantation in PD animal models has shown a certain degree of benefit,^58, 59, 60, 61^ additional studies are required to elucidate its clinical efficacy and safety. To date, one report has verified that human parthenogenetic stem cell-derived NSCs (hpNSCs) can successfully engraft, survive long-term and increase dopamine levels in the brains of rodent and nonhuman primate models of PD. In addition, hpNSCs have negligible tumourigenic potential and are safe for clinical application, thus supporting the approval of an hpNSC-based phase I/IIa study for the treatment of PD.^62, 63^

AD is characterized by increased levels of both soluble and insoluble amyloid beta peptides.^64^ It has also been reported that hippocampal neuronal mitochondria levels are decreased in AD patients. Transplantation of exogenous NSCs into transgenic AD mice leads to a significant increase in the number of mitochondria and the expression of mitochondria-related proteins, as well as improvements in mouse cognitive function.^65^ The therapeutic effect of NSC transplantation can be further improved by combining with cerebrolysin controlling amyloid precursor protein metabolism,^66^ self-assembled peptides providing a protective niche^67^ and nerve growth factor nanoparticles.^68^ Human NSCs have also been intensively investigated as an AD treatment in transgenic animal models.^69, 70^ Although many positive results have been reported based on those models, there were still negative consequences emerged. Marsh *et al.*^71^ used an immune-deficient AD model to examine the long-term effects of the transplantation of human NSC products and found that five months after transplantation, human NSCs had engrafted and migrated throughout the hippocampus; however, changes in brain-derived neurotrophic factor expression and increases in synaptic density were not observed. The disappointing result of this assay might be due to the failure of the human NSCs to terminally differentiate, which reinforces the notion that candidate cells need to be thoroughly evaluated for safety and efficacy before every transplantation. Considering these inconsistent data, to date, there have been no clinical study of NSC transplantation in AD patient.

HD is an autosomal-dominant inherited disease that induces caudate nucleus atrophy and is characterized by involuntary choreic movements, cognitive impairment and emotional disturbance.^72^ Animal HD models can be established by injecting quinolinic acid into rodent and primate striatum to simulate excitotoxicity. Human fetal NSCs can differentiate into neurons and astrocytes following transplantation into the rat striatum, partially eliciting behavioral and anatomical recovery in HD rats.^73^ Furthermore, intracerebral transplantation of NSCs combined with trehalose has been found to not only alleviate polyglutamine aggregation formation and decrease striatal volume but also extend lifespan in transgenic mouse model of HD.^74^ To further promote the beneficial effects of transplanting NSCs in animal models of HD, the timing of transplantation and cell preparation, NSC activity and co-transplantation of NSCs with helper cells, such as MSCs that secrete brain-derived neurotrophic factor, need to be fully considered based on the pathological conditions.^75, 76^

### Spinal cord injury

Spinal cord injury (SCI) is a severe physical injury and often gives rise to severe loss of motor function and secondary damage.^77^ There are no effective conventional treatments for SCI, but transplantation of NSCs in a mouse model of SCI leads to significant improvements in motor function recovery, thus indicating that NSCs can survive *in vivo*, differentiate, and alter the microenvironment of early chronic injury sites.^78^ In a primate SCI model, transplanted NSCs have been found to differentiate into cells expressing neuronal markers, thereby improving hind limb performance.^79^ Considering that treatment of SCI with NSCs is regulated by a variety of cytokines and proteins, drugs that modulate these factors will be a helpful adjunctive therapy, such as etanercept having anti-inflammatory and anti-apoptotic effects,^80^ free radical scavenger edaravone,^81^ and erythropoietin.^82^ With the rapid development of tissue engineering techniques, biomaterials have gradually been applied to SCI treatment and have provided new prospects for NSC transplantation; for example, biodegradable scaffolds with aligned columns^83^ and gelatine sponge scaffold.^84^ In contrast to these positive results, Anderson *et al.*^85^ found that human CNS-derived stem cells failed to show preclinical efficacy in a pathway study of cervical SCI. In that study, no evidence of donor cell differentiation into the neuronal lineage was observed. This failure might be attributed to the insufficient characterization of the clinical cell line supplied by the sponsor using potency assays. However, in a phase I/IIa clinical trial on the transplantation of fetal cerebral NSCs into 19 traumatic cervical SCI patients, 17 patients regained sensorimotor function after 1 year, and 2 patients showed complete motor but incomplete sensory recovery. There was no evidence of cord damage; syrinx or tumor formation; neurological deterioration; and exacerbating neuropathic pain or spasticity.^86^

### Stroke

Stroke is an acute cerebrovascular disease that includes ischemic and hemorrhagic stroke.^87, 88^ Transplanted mouse iPSC-derived or human fetus-derived NSC lines have been reported to provide neurotrophic factors and increase angiogenesis and neurogenesis in both ischemic and hemorrhagic stroke animal models.^89, 90^ Besides, Li *et al.*^91^ demonstrated that compared with transplantation of NPCs alone, co-transplantation with vascular progenitor cells, which might support NPC survival, leads to more effective improvements in neurovascular recovery and attenuation of the infarct volume. Furthermore, the application of three-dimensional electrospun fibers as cell carriers has shown promising results and has been found to extend the survival rate of administered human NSCs by blocking microglial infiltration in an animal model of stroke induced by middle cerebral artery occlusion.^92^ Despite positive results from animal assays, caution should be exercised before clinical trials, because other findings have suggested that endogenous neurogenesis is decreased after NPC treatment via microglial activation.^93^ However, in pioneering work, the United Kingdom has already initiated phase I/II clinical trials on the treatment of ischemic stroke with CTX0E03, an immortalized human NSC line.^94^ Thirteen men were recruited for the phase I trial in which a single dose of up to 20 million cells was implanted via stereotactic ipsilateral putamen injection, and whereas neurological function was improved, no adverse events were observed. Based on the results of the phase I trial, the phase II trial was initiated and is still underway.

### Traumatic brain injury

The principal mechanisms of traumatic brain injury (TBI) are classified as focal brain damage and diffuse brain damage, which correspond to contact injury types and acceleration/deceleration injury types.^95^ TBI is extremely likely to cause cognitive and memory deficits as well as motor impairments. Previous study concluded that NSCs may stabilize the cortical microenvironment after TBI.^96^ Transplantation of mouse brain-derived NSCs into brain injury mice effectively prevents astroglial activation and microglial/macrophage accumulation while increasing oligodendrocytes and repairing and maintaining normal neuron function.^97^ A sodium hyaluronate collagen scaffold loaded with bFGF promotes the survival and differentiation of transplanted rat NSCs and promotes functional synapse formation to repair traumatic brain injuries in rats.^98^ In a clinical study, Zhu’s group labeled autologous cultured NSCs with superparamagnetic iron oxide nanoparticles and then stereotactically implanted them around the regions of brain trauma in TBI patients. Magnetic resonance imaging tracking images showed the accumulation and proliferation of cells around the lesion and even their migration from the primary sites of injection to the border of the damaged tissue.^99^

### Epilepsy

Epilepsy is an abnormal discharge of cerebral neurons resulting from an imbalance between excitation and inhibition in the CNS; this imbalance leads to transient cerebral dysfunction, which is mainly related to changes in ion channel neurotransmitters and glial cells. Nearly 30% of patients with temporal lobe epilepsy (TLE) are resistant to antiepileptic drugs. Current reports demonstrate that NSC transplantation can inhibit spontaneous seizures. When transplanted into the epileptic sites of the hippocampus, exogenous NSCs produce a specific type of neuron that synthesizes the inhibitory neurotransmitter *γ*-aminobutyric acid and astrocytes that secrete anticonvulsant factors, which slow the cognitive and emotional dysfunction caused by TLE.^100, 101^

### Cerebral palsy

Cerebral palsy (CP) is a group of permanent movement disorders that appear in early childhood, which is due to the formation of non-progressive lesions in the developing central nervous system.^102^ At present, CP treatment includes many measures, but none of these treatments can cure CP patients. In recent years, the safety and efficacy of NSC/NPC therapy for CP has been evaluated. Based on current studies, transplantation of NSCs transduced with vascular endothelial growth factor can partially slow brain damage and has neuroprotective effects on neonatal rats with hypoxia-mediated CP, thus suggesting a new potential strategy for CP treatment.^103, 104^ In clinic, motor development and cognition have been found to be significantly accelerated after aborted fetal tissue-derived NPCs were injected into the lateral ventricle of children with CP.^105^ Besides, CP patients undergo bone marrow MSC-derived neural stem-like cell transplantation were followed up for long term. Improvements in motor function have been observed following this procedure but not improvements in language quotient, demonstrating that neural stem-like cells derived from autologous MSCs may be a better cell type for CP therapy.^106^

### Neonatal hypoxic-ischemic encephalopathy

Hypoxic-ischemic encephalopathy (HIE) is a condition that occurs when the entire brain is incompletely deprived of adequate oxygen supply.^107, 108^ Recent research has shown that mild hypothermia therapy for neonatal encephalopathy via NSC transplantation can attenuate disease progression and prevent long-term damage associated with HIE.^109^ The ginsenoside Rg1 has been reported to be effective in promoting the recovery of brain function after injury, and thus, Li *et al.*^110^ performed ginsenoside-induced NSC transplantation into HIE rats and reported improved behavioral capacity compared with the control group that received only saline, thus presenting a promising new treatment for brain injury. In addition, human embryonic NSC treatment significantly improves learning, memory and cognitive deficits in HIE rat.^111^ Based on these preclinical studies, much effort is required to translate the NSC transplantation techniques for evaluation in clinical trials involving HIE patients.

### Other disorders

Age-related macular degeneration (AMD) is characterized by a progressive loss of photoreceptors, and it is the leading cause of vision loss.^112^ Although treatment options for AMD are limited, transplantation of human NPCs into a rodent model of retinal degeneration not only halted the progression of vision loss but also preserved photoreceptors and visual function long term.^113, 114^ A phase I/II clinical study is investigating the safety and preliminary efficacy of unilateral subretinal transplantation of human NSCs in subjects with geographic atrophy secondary to AMD. In addition, intensive research and development have resulted in a series of NSC products and genetically modified NSCs entering the preclinical study phase and clinical testing phase for the treatment of malignant and non-malignant diseases, such as recurrent high-grade gliomas, metastatic encephaloma and lower limb ischemia (Tables 2 and 3).

## Conclusions and Perspectives

NSCs show great plasticity in the treatment of nervous system diseases and can be induced to differentiate into mature neural cells of various types, including neurons, astrocytes and oligodendrocytes. Under defined conditions, NSCs have a broad differentiation repertoire and can even give rise to non-ectodermal cells, including hematopoietic cells and skeletal myogenic cells.^115, 116, 117^ In addition, NSCs secrete neurotrophic factors that improve the lesion microenvironment, thereby generating appropriate conditions for pathological repair. The above findings confirm the significant value of NSCs in basic research and in therapeutic applications. The therapeutic effect of NSC transplantation can be further improved with adjuvant pharmaceutical agents, genetic modification, three-dimensional grafts or helper cells, which may improve cell survival and differentiation into specific types of cells.

Initially, NSCs could be obtained only from embryonic brains, and therefore, the limited source, technical issues in isolation and low purity blocked the progression of NSC-based cell therapy. In addition, there were ethical and moral concerns. Currently, emerging technologies continuously improve the methods that can be used to obtain NSCs, especially the identification of ESCs and the development of iPSCs, thus enabling the acquisition of a large number of NSCs and promoting basic research on NSCs. However, the differentiation of pluripotent stem cells into NSCs with high purity usually requires a long time and is accompanied by safety issues, thus complicating the translation of this procedure into a clinical therapy. Somatic cell transdifferentiation, especially methods without a viral strategy or integration of exogenous genes such as chemical-, growth factor-, 3D culture- or microRNA-induced cell transdifferentiation,^118^ into NSCs avoids the previous shortcomings and provides a very attractive strategy for the mass production of NSCs for clinical application.

**Figure 3:**
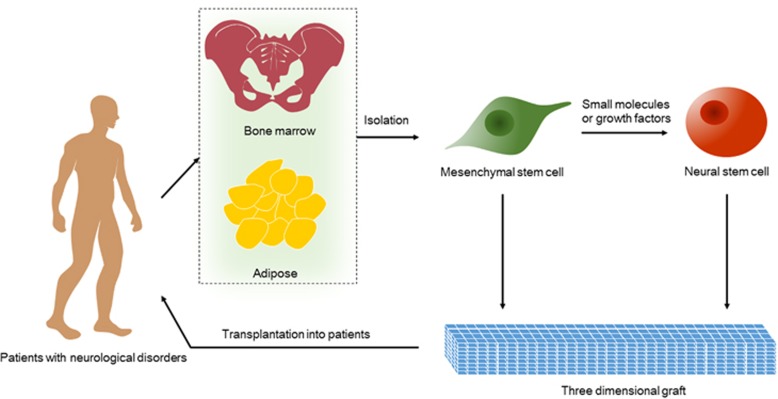
Therapeutic strategies for the derivation and transplantation of NSCs. MSCs are ideal for NSC derivation because of the ease of accessibility from patient bone marrow and especially subcutaneous adipose tissue. NSCs transdifferentiated from plastic MSCs via non-viral and non-genetic methods, such as induction with small molecules or growth factors, are likely safer for clinical application. Transplantation of derived NSCs or co-transplantation of both cells with or without three-dimensional grafts into patents is dependent on disease-specific targets

MSCs have great value in regenerative medicine because autologous MSCs are easily harvested and can be effectively induced into a variety of specialized cells, including neural cells. MSC-derived neural stem-like cells have been found to exhibit significant neuroprotective effects.^119, 120^ In addition, MSCs release paracrine signals that enhance neuronal cell proliferation and the differentiation of human NSCs *in vitro* in co-culture systems, except for the direct transition of MSCs to neural stem-like cells.^121, 122, 123^ Moreover, MSCs are a type of immune cell, which can be applied to decrease immune rejection, prolong the survival time of grafts and treat immune dysregulation. Importantly, MSCs alone have been used in many clinical trials for the treatment of neurodevelopmental disorders.^59, 124, 125^ Co-transplantation of bone marrow MSCs and adult NSCs in a transgenic rat model of HD has been found to confer long-term behavioral benefits and to improve survival of the transplanted NSCs.^126^ Thus, it should be anticipated that MSC-derived NSC-based therapy with or without MSCs will be a major direction for the treatment of nerve diseases in the future (Figure 3).

Although NSC treatment has exhibited some success in a variety of animal disease models, many problems remain to be addressed before transition to clinical applications because of the substantial physiological differences between humans and animals. First, clinical treatments must abide by standardized protocols. Therefore, detailed and efficient standards must be established for therapeutic routines, including stem cell types, the time of transplantation and cell dosage. Purity of NSCs is the priority and needs to be addressed and approved, as contamination of other cells may cause unexpected side effects. The optimal transplantation time for NSCs should be evaluated for each type of acute or chronic disease. Along with others, we have found that even primary tissue-derived NSCs or NPCs can form a clot *in vivo* if transplanted at high density; thus, the density of transplanted NSCs must be precisely controlled to avoid secondary damages to the injected tissues. Therapeutic routines for NSC administration, including local injection via intracranial or intraspinal routes and systemic injection via intravenous or intrathecal routes, are highly dependent on the lesion site. Second, stem cells that are used as seeds must be verified as safe both *in vitro* and *in vivo*. To accomplish this, we suggest carrying out deep sequencing and checking tumor formation potency in mice for every lot of manufactured NSC products. Third, the poor survival rate and the modest treatment effects of NSCs *in vivo* are major problems that still remain to be solved. Furthermore, the underlying mechanisms of stem cell therapy are still unclear, and hence, additional studies are required. Further basic research in related areas may help resolve the above-mentioned issues. Finally, advanced imaging techniques are required to monitor the physiological state of transplanted NSCs *in vivo* to exclude tumourigenicity and other pitfalls.

## Publisher’s Note

Springer Nature remains neutral with regard to jurisdictional claims in published maps and institutional affiliations.

## Figures and Tables

**Table 1:** Derivation of neural stem cells

**Species**	**Original tissues/cells**	**Treatment**	**Duration to derive NSCs**	**Refs**
*Isolation from primary tissues*				
Mouse	Striatum	EGF	1 week	^1^
Mouse	Thoracic spinal cord	EGF, bFGF	1 week	^3^
Mouse	Dentate gyrus, SVZ	EGF, bFGF	1–3 weeks	^4, 10, 11^
Mouse	Periventricular region	EGF, bFGF, heparin	2 weeks	^5^
Human and rat	Periventricular region	EGF, bFGF, heparin	2–3 weeks	^6^
Mouse	Olfactory bulb	EGF, bFGF	1–2 weeks	^7^
Human	Olfactory bulb	EGF, bFGF	1–2 weeks	^8^
Mouse	Postnatal cerebellum	EGF, bFGF	2 weeks	^9^
				
*Differentiation from pluripotent stem cells*				
Human	ESCs	Suspension culture	3–4 weeks	^13^
Human	ESCs	Adhesion co-culture with stromal cells MS-5	3 weeks	^18^
Human	ESCs	Adherent monolayer culture	3–5 weeks	^20^
Human	iPSCs	Suspension and adherent culture	2–5 weeks	^21^
				
*Transdifferentiation from somatic cells*				
Mouse	Fibroblasts	*Oct4, Sox2, Klf4, c-Myc*	2–3 weeks	^24, 25^
Mouse	Fibroblasts	*Brn4/Pou3f4, Sox2, Klf4, c-Myc, E47/Tcf3*	4–5 weeks	^26^
Mouse	Fibroblasts	*Foxg1, Sox2, Brn2*	3–4 weeks	^27^
Mouse and human	Fibroblasts	*Sox2*	2–3 weeks	^28^
Human	Fibroblasts	*ZFP521*	3–4 weeks	^29^
Primate	Fibroblasts	*OCT4, SOX2, KLF4, c-MYC*, SB431542, CHIR99021	2–3 weeks	^30^
Human	Fibroblasts	*OCT4,* A83-01, CHIR99021, NaB, LPA, Rolipram, SP600125	4 weeks	^31^
Mouse	Sertoli cells	*Ascl1, Ngn2, Hes1, Id1, Pax6, Brn2, Sox2, c-Myc, Klf4*	4–5 weeks	^32^
Mouse	Liver cells and B cells	*Brn2, Hes1, Hes3, Klf4, c-Myc, Notch1, PLAGL1, Rfx4*	4–5 weeks	^33^
Human	Urine cells	S*OX2, KLF4, c-MYC*, *SV40LT, miR302-367,* A83-01, PD0325901, CHIR99021, Thiazovivin, DMH1	4–5 weeks	^34^
Human	Astrocytes	*OCT4, SOX2, NANOG*	2–3 weeks	^35^
Human	Cord blood CD34^+^ cells	*OCT4*, CHIR99021	2–3 weeks	^36^
Mouse and human	Fibroblasts, urine cells	VPA, CHIR99021, Repsox	3 weeks	^37^
Mouse	Fibroblasts	CHIR99021, LDN193189, A83-01, Hh-Ag1.5, Vc, SMER28, RG108, Parnate	2 weeks	^38^
Mouse	Fibroblasts	VPA, Forskolin, Tranylcypromine, CHIR99021, Repsox, SB431542, Dorsomorphin	2 weeks	^39^
Mouse	Fibroblasts	VPA, A83-01, Purmorphamine, Vc, NaB, Thiazovivin	2 weeks	^40^
Mouse and human	MSCs	bFGF, EGF	1–2 weeks	^42, 43^
Mouse	Fibroblasts	bFGF, EGF, heparin, LIF	3–4 weeks	^45^
Mouse	Fibroblasts	3D sphere culture	NA	^46^

Abbreviations: bFGF, basic fibroblast growth factor; 3D, three dimension; EGF, epidermal growth factor; ESCs, embryonic stem cells; iPSCs, induced pluripotent stem cells; LIF, leukemia inhibitory factor; LPA, lysophosphatidic acid; MSCs, mesenchymal stem cells; NA, not available; NaB, sodium butyrate; SVZ, subventricular zone; Vc, ascorbic acid; VPA, valproic acid

**Table 2:** Treating neurological disorders in animal models via neural stem cell transplantation

**Disease target**	**Animal model**	**Transplanted cell source**	**Therapeutic mechanism**	**Outcome**	**Refs**
ALS	SOD1 (G93A) transgenic rat	Human fetal spinal cord-derived NSCs	Increased glial cell line-derived and brain-derived neurotrophic factors	Improved motor function and extended lifespan	^49^
ALS	SOD1 (G93A) transgenic mouse	Human iPSCs-derived NSCs	Increased neurotrophic factors and enhanced gliosis	Improved neuromuscular function and extended lifespan	^50^
PD	6-OHDA-induced mouse	NSCs transdifferentiated from mouse sertoli cells with Lmx1a	Enhanced tyrosine hydroxylase signal and increased endogenous dopaminergic neurons	Improved motor function	^57^
PD	MPTP-induced monkey	Human parthenogenetic stem cell-derived NSCs	Increased striatal dopamine concentration, fiber innervation and number of dopaminergic neurons	Promoted behavior recovery	^63^
AD	APP/PS1 transgenic mouse	Mouse fetal brain-derived NSCs	Enhanced mitochondria biogenesis	Decreased cognitive deficits	^65^
AD	APP transgenic mouse	Mouse cortical NSCs with cerebrolysin	Increased survival of grafted cells	NA	^66^
AD	A*β*-induced AD rat	Rat brain-derived NSCs with designer self-assemble peptide	Increased survival and differentiation of the grafted cells, enhanced neuroprotection, anti-neuroinflammatory and paracrine action	Improved behavior recovery	^67^
AD	192IgG-saporin-induced AD rat	Rat fetal brain-derived NSCs with nerve growth factor nanoparticles	Increased basal forebrain cholinergic neurons, hippocampal synapses and AchE-positive fibers	Improved spatial learning and memory	^68^
AD	3x and Thy1-APP transgenic mouse	Neprilysin-modified human NSCs	Decreased A*β* plaques and increased synaptic density and plasticity	Decreased Alzheimer's disease pathology	^69^
AD	APP/PS1 transgenic mouse	Human brain-derived NSCs	Enhanced neuronal connectivity and metabolic activity	Improved cognitive, learning and memory, no change in anxiety level	^70^
AD	Rag-5xfAD transgenic mouse	Commercial human fetal brain-derived CNS-SCs	No changes in brain-derived neurotrophic factor and no increase in synaptic density	Fail to improve learning and memory	^71^
HD	R6/2 transgenic mouse	C17.2 NSCs with trehalose	Decreased ubiquitin-positive aggregation, polyglutamine aggregation and striatal volume	Improved motor function, memory performance and survival rate	^74^
SCI	Weight drop on mouse	Commercial human fetal brain-derived CNS-SCs	Increased oligodendrocytes and neurons	Improved locomotor recovery	^77^
SCI	Weight drop on primate	Adult monkey NSCs	Migration of NSCs to the injury sites	Improved hind limb performance	^79^
SCI	Hemisection of rat	Rat fetal brain-derived NSCs with etanercept	Anti-inflammation and anti-apoptosis	Re-myelination, neural regeneration and improved locomotor function	^80^
SCI	Weight drop on rat	Rat fetal brain-derived NSCs with edaravone	Decreased oxidative damage, increased survival and differentiation of NSCs	Improved rear-limb function	^81^
SCI	Hemisection of rat	Rat fetal brain-derived NSCs with biodegradable scaffolds	Improved axonal regeneration	No functional recovery	^83^
SCI	Hemisection of rat	Rat brain-derived NSCs-modified by NT-3 and TrkC gene with gelatin sponge scaffold	Increased survival of axotomized neurons and axonal regeneration	Improved partial locomotor functional recovery	^84^
SCI	Weight drop on mouse	Commercial human fetal brain-derived CNS-SCs	No neuronal lineage differentiation of donor cells	No functional recovery	^85^
Stroke	MCAO in rat	iPSC line-derived NPCs	Enhanced endogenous neurogenesis and angiogenesis and increased trophic factors	Improved functional recovery	^89^
Stroke	MCAO in rat	Mouse fetal brain-derived NSCs and and ESCs-derived vascular progenitor cells	Enhanced neurovascular recovery and neurotrophic factors and decreased infarct volume	Improved functional neurological deficits	^91^
Stroke	MCAO in rat	Sliding fibers containing human brain-derived NSCs	Increased survival rate of administered NSCs and decreased microglial infiltration	NA	^92^
TBI	CCI in mouse	Mouse brain-derived NSCs	Increased oligodendrocytes, decreased astroglial activation and microglial/macrophage accumulation	Delayed spatial learning deficits	^96^
TBI	CCI in rat	Sodium hyaluronate collagen scaffold loaded with rat brain-derived NSCs and bFGF	Increased survival and differentiation of NSCs and enhanced functional synapse formation	Improved cognitive function recovery	^98^
Epilepsy	Kainic acid-induced rat	Rat embryonic medial ganglionic eminence-derived NSCs	Increased GABAergic neurons and GDNF expression in hippocampal astrocytes	Reduced spontaneous recurrent motor seizures	^101^
CP	UCAO plus hypoxia in rat	Rat fetal NSCs transfected with VEGF	Increased VEGF protein expression and decreased neuronal apoptosis	Improved spatial discrimination, learning, memory recall capabilities and locomotor function	^103^
CP	UCAO plus hypoxia in rat	Rat fetal NSCs transfected with VEGF	Increased VEGF protein expression and neuroprotection	Improved motor function	^104^
HIE	UCAL in neonatal mouse	Mouse fetal brain-derived NSCs with mild hypothermia treatment	Increased survival rate of NSCs, decreased caspase-3, NF-*κ*B and cerebral infarct volumes	Improved functional recovery	^109^
HIE	UCAL in neonatal rat	Human fetal brain-derived NSCs with ginsenoside Rg1	Enhanced latency of somatosensory evoked potentials and increased neurotrophic factors	Improved learning and memory behavior	^110^
HIE	Unilateral carotid artery cutting in rat	Human embryonic NSCs	Decreased IL-1*β* expression, increased NF-*κ*B translocation, reduced brain tissue loss and white matter injury	Alleviated sensorimotor disabilities, improved learning, memory, and cognitive functions	^111^

Abbreviations: A*β*, amyloid beta; AD, alzheimer’s disease; ALS, amyotrophic lateral sclerosis; APP, amyloid precursor protein; CCI, controlled cortical impact; CNS-SCs, central nerve system stem cells; CP, cerebral palsy; C17.2 cells, a murine neural progenitor cell line; ESCs, embryonic stem cells; GDNF, glial cell-derived neurotrophic factor; HD, huntington's disease; HIE, neonatal hypoxic-ischemic encephalopathy; IL-1*β*, interleukin-1*β*; iPSCs, induced pluripotent stem cells; MCAO, middle cerebral artery occlusion; MPTP, 1-methyl-4-phenyl-1,2,3,6-tetrahydropyridine, used as neurotoxins; NA, not available; NF-*κ*B, nuclear factor-*κ*B; NPCs, neural progenitor cells; NSCs, neural stem cells; 6-OHDA, 6-hydroxydopamine, used as neurotoxins; PD, parkinson's disease; PS1, presenilin 1; Rag-5xfAD mice, an immune-deficient transgenic model exhibited several hallmarks of AD pathogenesis; SCI, spinal cord injury; SOD1, superoxide dismutase 1; TBI, traumatic brain injury; UCAL, unilateral carotid artery ligation; UCAO, unilateral carotid artery occlusion; VEGF, vascular endothelial growth factor

**Table 3:** Clinical trials for neural stem cell transplantation

**Conditions**	**Transplanted cells**	**Status/intervening results**	**Phase**	**Location**	**Start year**
ALS	Spinal cord-derived NSCs	Safe with unilateral and bilateral intraspinal lumbar microinjection	Phase I	United States	2011
ALS	Spinal cord-derived NSCs	No study results	Phase II	United States	2012
ALS	Fetal brain-derived NSCs	Improved tibialis anterior	Phase I	Italy	2012
ALS	CNS10-NPC-GDNF	Recruiting	Phase I	United States	2016
PD	Parthenogenetic stem cell-derived NSCs	Recruiting	Phase I	Australia	2015
PD	ESCs-derived NPCs	Recruiting	Phase I/II	China	2017
PD	Fetal brain-derived NSCs	Invitation	Phase II/III	China	2017
MS	MSCs-derived NPCs	Active, not recruiting	Phase I	United States	2013
SCI	Fetal brain-derived NSPCs	Safe and well-tolerated	Phase I/II	Korea	2005
SCI	MSCs-derived NSCs	Active, not recruiting	Phase I/II	Russia	2014
SCI	Spinal cord-derived NSCs	Recruiting	Phase I	United States	2013
SCI	CNS stem cells	Terminated, no study results	Phase II	United States, Canada	2014
SCI	NSCs combined with Scaffold	Recruiting	Phase I/II	China	2016
SCI	CNS stem cells	No study results	Phase I/II	Canada, Switzerland	2011
Stroke	CTX0E03	Improved neurological function with no adverse events	Phase I	United Kingdom	2010
Stroke	CTX0E03	Active, not recruiting	Phase II	United Kingdom	2014
CP	Fetal brain-derived NPCs	Improvement of functional development and no delayed complications	NA	China	2005
CP	Fetal brain-derived NSCs	Improvement with varying degrees and no severe adverse reactions	NA	China	2005
CP	Bone marrow MSCs- derived NSCs-like cells	Optimal improvement in motor function	NA	China	2010
CP	NSCs	Active, not recruiting	NA	China	2016
HIE	NPCs with paracrine factors from MSCs	Recruiting	NA	China	2014
MD	CNS stem cells	No study results	Phase I/II	United States	2012
LLI	CTX0E03	Active, not recruiting	Phase I	United Kingdom	2013
Glioma	NSCs expressing *E. Coli CD*	No study results	Phase I	United States	2010
Glioma	NSCs expressing *E. Coli CD*	Recruiting	Phase I	United States	2013
Glioma	NSCs expressing Carboxylesterase	Recruiting	Phase I	United States	2014
Glioma	NSCs loaded with oncolytic adenovirus	Recruiting	Phase I	United States	2017
GBM	NPCs	Active, not recruiting	Phase I	United States	2011
IBM	NSCs	Terminated, no study results	Phase III	United States	2007

Abbreviations: ALS, amyotrophic lateral sclerosis; CNS10-NPC-GDNF, human neural progenitor cells secreting glial cell line-derived neurotrophic factor; CNS, central nerve system; CP, cerebral palsy; CTX0E03, immortalized human neural stem cell line; ESCs, embryonic stem cells; GBM, glioblastoma; HIE, neonatal hypoxic-ischemic encephalopathy; IBM, Intraparenchymal brain metastases; LLI, lower limb ischemia; MD, macular degeneration; MS, multiple sclerosis; MSCs, mesenchymal stem cells; NPCs, neural progenitor cells; NSCs, neural stem cells; NSPCs, neural stem/progenitor cells; PD, parkinson's disease; SCI, spinal cord injury
